# Association between gastric Candida colonization and surgical site infections after high-level hepatobiliary pancreatic surgeries: the results of prospective observational study

**DOI:** 10.1007/s00423-020-02006-7

**Published:** 2020-10-15

**Authors:** Kazuyuki Gyoten, Hiroyuki Kato, Aoi Hayasaki, Takehiro Fujii, Yusuke Iizawa, Yasuhiro Murata, Akihiro Tanemura, Naohisa Kuriyama, Masashi Kishiwada, Shugo Mizuno, Masanobu Usui, Hiroyuki Sakurai, Shuji Isaji

**Affiliations:** Department of Hepatobiliary Pancreatic and Transplant Surgery, School of Medicine, Mie University, 2-174 Edobashi, Tsu, Mie 514-0001 Japan

**Keywords:** Candida, Gastric juice, Hepatobiliary pancreatic surgeries, Surgical site infections

## Abstract

**Aim:**

High-level hepatobiliary pancreatic (HBP) surgeries are highly associated with surgical site infections (SSIs), in which microorganisms have a significant role. In the present study, we investigated whether gastric Candida colonization had a significant role in SSIs after high-level HBP surgeries.

**Methods:**

Between May 2016 and February 2017, the 66 patients who underwent high-level HBP surgeries were enrolled in the present study. The gastric juice was prospectively collected through nasogastric tube after general anesthesia induction and was incubated onto the CHROMagar Candida plate for the cultivation of various *Candida* species. First of all, we compared the incidence of SSIs according to the presence or absence of *Candida* species in gastric juice. Secondly, we evaluated the variables contributing to the development of SSIs by multivariate analysis. The protocol was approved by the medical ethics committee of Mie University Hospital (No.2987).

**Results:**

Gastric Candida colonization was identified in 21 patients (group GC) and was not identified in the other 45 patients (group NGC). There were no differences in preoperative variables including compromised status, such as age, nutritional markers, complications of diabetes mellitus, and types of primary disease between the two groups. SSIs occurred in 57.1% (12/21) of group GC and in 17.8% (8/45) of group NGC, showing a significant difference (*p* = 0.001). Multivariate analysis revealed gastric Candida colonization as a significant risk factor of SSIs (OR 6.17, *p* = 0.002).

**Conclusion:**

Gastric Candida colonization, which is not a result of immunocompromised status, is highly associated with SSIs after high-level HBP surgeries.

**Trial registration:**

Japan Primary Registries Network; UMIN-CTR ID: UMIN000040486 (retrospectively registered on 22nd May, 2020).

**Electronic supplementary material:**

The online version of this article (10.1007/s00423-020-02006-7) contains supplementary material, which is available to authorized users.

## Introduction

High-level hepatobiliary pancreatic (HBP) surgeries such as major hepatectomy and pancreaticoduodenectomy (PD) are associated with a high incidence of morbidity and mortality despite advanced surgical techniques and postoperative care [1, 2]. A large portion of the morbidity was constituted by postoperative infectious complications: pancreatic fistula, biliary fistula, intra-abdominal abscess, and wound infection [3–6]. Several reports have focused on the association between microorganisms and the postoperative infections, in which bacteriobilia caused by preoperative biliary drainage was significantly associated with wound infection, intra-abdominal abscess, and sepsis [7–11]. In such cases, we have focused on the association between Candida colonization and postoperative complications.

Candida colonization in the human gastrointestinal tract is generally recognized as inapparent infection but sometimes develops to Candida infection of candidemia and Candida peritonitis in patients recovering from abdominal surgeries, in which *Candida* species invade from mucosal defects and the amount of *Candida* species increases in non-functioning bowels [12, 13]. Recent reports revealed that the presence of a high amount of *Candida* species is related to invasive Candida infection and inflammatory disease, which implies that Candida colonization might have a negative influence on the clinical course [14, 15]. Previously, we clarified that biliary candidiasis could be the independent cause of surgical site infections (SSIs) after PD, in which biliary candidiasis was recognized as inapparent infection from the upper gastrointestinal tract to hepaticojejunostomy [10, 11]. Bacterial and fungal species in the upper gastrointestinal tract may have a significant impact on postoperative infectious complications. To date, however, there have been no reports regarding the impact of gastric Candida colonization on postoperative infectious complications in high-level HBP surgeries.

In the present study, we aimed to clarify the influence of gastric Candida colonization on SSIs in patients who underwent high-level HBP surgeries.

## Methods

### Patients

Between May 2016 and February 2017, the 66 patients who underwent high-level HBP surgeries at Mie University Hospital had been prospectively registered for the current study after obtaining informed consent. High-level HBP surgeries were defined as the following operative procedures: pancreaticoduodenectomy, distal pancreatectomy for pancreatic ductal adenocarcinoma (PDAC), total pancreatectomy, major hepatectomy of three segments or more, anatomical sectionectomy and subsectionectomy, common bile duct resection for congenital biliary disease, and liver transplantation according to the definition of the Japanese Society of Hepato-Biliary-Pancreatic Surgery (JSHBPS) [16]. Operations were performed or supervised by board-certified expert surgeons of the JSHBPS.

The study protocol was approved by the medical ethics committee of Mie University Hospital (No. 2987), and the study was performed in accordance with the ethical standards established in the 1964 Declaration of Helsinki.

### Surgical procedures and perioperative administration

In PD, the reconstruction was carried out by using a modified child method: end-to-side pancreaticojejunostomy, end-to-side hepaticojejunostomy, and end-to-side gastrojejunostomy. In pancreaticojejunostomy, we used a pair-watch suturing technique, allowing us to standardize the anastomosis regardless of the pancreatic texture and the diameter of the main pancreatic duct (MPD) [17]. A 5Fr pancreatic stent tube was usually inserted in cases with a soft pancreas, narrow MPD, and/or severe comorbidity. In cases of the hepaticojejunostomy, instead, we routinely inserted the 5Fr biliary stent tube, which was guided externally through the jejunal loops. In distal pancreatectomy, the pancreatic parenchyma was transected with an ultrasonic dissector, the MPD was ligated, and the pancreas stump was sutured by fish mouth closure. In hepatectomy, a cavitron ultrasonic suction aspirator (CUSA; Valleylab, Boulder, NY) was used for parenchymal dissection together with a monopolar soft-coagulation device.

Before abdominal closure, intra-abdominal irrigation using 3- to 5-l saline was conducted. Before skin closure, prophylactic wound irrigation was performed to avoid SSIs. As prophylactic antimicrobial therapy, flomoxef sodium (an oxacephem antibiotic) of 1 g was administered every 3 h during operation, and it was administered every 12 h between postoperative days one and three. When flomoxef-resistant bacteria were identified in the bile obtained by preoperative biliary drainage, specific antibiotics were administered based on the culture results. Antifungal treatments were not performed regardless of gastric Candida colonization. A 19Fr closed suction drain was placed in Winslow’s foramen or at the cut end of the pancreas and the liver.

### Culture of gastric juice and diagnosis of gastric Candida colonization

All patients had 250 ml of an 18% carbohydrate-rich beverage (Arginaid Water: Nestle Health Science Co., Tokyo, Japan) at 3 h before general anesthesia to reduce the risk of developing postoperative insulin resistance as part of the enhanced recovery after the surgery protocol [18]. Immediately after general anesthesia induction, a nasogastric tube (16Fr) was inserted by an anesthesiologist, and 5 ml of gastric juice was collected and transferred to a 10-ml-sized sterile bottle. The gastric juice was placed directly on one quadrant of the CHROMagar Candida plate (Kanto Chemical Co, Inc., Tokyo, Japan) using an inoculation loop for the cultivation of fungal species and try/soy blood agar (sheep) No. 2 (Kyokuto Pharmaceutical Industrial Co. Ltd., Tokyo, Japan) for the cultivation of bacterial species.

The microbiological growth was checked every 24 h over 72 h of incubation. After 72 h of incubation, the microbiological growth was classified according to the growth area of the plate: no growth, suspecting contamination, or false positive (less than one-quadrant growth) and positive (more than one-quadrant growth). No gastric Candida colonization (NGC) was defined as no growth or less than one-quadrant growth, and gastric Candida colonization (GC) was defined as more than one-quadrant growth. *Candida* species were determined according to the colors of the colonies: *Candida albicans* in green, *Candida glabrata* in purple, *Candida tropicalis* in blue, *Candida parapsilosis*, and *Candida krusei* in pink.

### Evaluations of risk factors for gastric Candida colonization

We selected the clinical variables from pre- and intraoperative factors that may influence the development of gastric Candida colonization. Preoperative factors included age, gender, body mass index (BMI), performance status (PS), the presence of cachexia, primary disease (malignancy or not), history of broad specific antibiotics, preoperative biliary drainage, chemotherapy, chemoradiotherapy, administration of proton pump inhibitor (PPI) or histamine-2 (H2) receptor blocker including the dosage, use of total parenteral nutrition, therapeutic use of glucocorticoid, diabetes mellitus, hypertension,dyslipidemia, smoking (Brinkman index of more than 400), alcohol consumption (ethanol consumption of more than 60 g per day), history of other malignant diseases, disease types, peripheral blood values of white blood cells, hemoglobin, platelet, C-reactive protein, albumin, neutrophil/lymphocyte ratio[19]; and prognostic nutritional index [20]. Intraoperative factors included operative procedure, operative duration, intraoperative blood loss, and blood transfusion. The diagnostic criterion for cachexia was weight loss greater than 5%, or weight loss greater than 2% in individuals already showing depletion according to current body weight and height (BMI < 20 kg/m^2^) or sarcopenia [21]. Sarcopenia was diagnosed according to the algorithm of the Asian Working Group for Sarcopenia 2019: handgrip strength < 28 kg for men and < 18 kg for women; 6-m walk < 1.0 m/s ; cutoff values for muscle mass measured by bioimpedance < 7.0 kg/m2 in men and < 5.7 kg/m2 in women [22].

### Definitions of postoperative complications and risk factor analyses

The primary endpoint was to evaluate whether gastric Candida colonization affects the incidence of SSIs after surgery. In accordance with the Centers for Disease Control and Prevention (CDC) guidelines, SSIs were categorized as incisional SSIs and organ or space SSIs which involved any part of the internal organs other than the incision that is treated with the surgical procedure or interventional approach [23]. Secondary endpoints were to evaluate the occurrence of Clavien–Dindo (C-D) grade IIIa or higher postoperative complications [24], duration of intensive care unit (ICU) stay, duration of hospital stay, and the readmission rate until 180 days after surgery. The postoperative pancreatic fistula was defined according to the International Study Group on Pancreatic Fistula [3]. The postoperative biliary fistula was defined as the presence of bile or bile-stained fluid from surgical drains after postoperative day three.

### Statistical analysis

The sample size was based on previous studies, in which 28 patients underwent PD [11]. SSIs occurred in 5 (55%) of 9 patients with gastric Candida colonization and in 4 (21%) of 19 without gastric Candida colonization. Assuming an α level of 0.05 and 80% power, the required number of patients for each group to observe SSIs difference of 34% was 31. To allow for a 10% drop-out rate, around 70 patients were set as a sample size. All continuous values were presented as median with range. Continuous variables were compared using Student’s *t* test or the Mann–Whitney test according to the data distribution with or without normality. Categorical variables were compared using Pearson’s chi-squared test. Stepwise forward multiple logistic regression analysis of factors contributing to postoperative complications was carried out. Statistical data analysis was performed using the SPSS program, version 24.0 (SPSS, Chicago, Ill, USA). A *p* value less than 0.05 was considered statistically significant.

## Results

Of the 70 patients originally enrolled in the current study, 4 were excluded as subjects: two died of non-infectious complications within 5 days of the operation, and the other two had too little gastric juice to perform a fungal and bacterial culture. Therefore, the total number of subjects was 66 patients. Table 1 shows the background of the subjects including pre- and intraoperative variables. There were 41 males and 25 females. Their median age was 69 years (23–91 years). Their preoperative diagnoses were PDAC in 30 patients, intraductal papillary mucinous neoplasm (IPMN) in 8, biliary tract cancer (BCD) in 10 (distal cholangiocarcinoma in 5, perihilar cholangiocarcinoma in 3, ampullary carcinoma in 1, and intrahepatic cholangiocarcinoma in 1), hepatocellular carcinoma in 6, and the other diseases in 12 (liver metastasis from colon cancer in 2, congenital biliary dilatation in 2, refractory cholangitis after operation for congenital biliary dilatation in 2, hepatectomy for living donor liver transplant in 2, intraductal papillary neoplasm of the bile duct in 1, refractory cholangitis after Kasai’s operation for biliary atresia in 1, papillary adenoma of the ampulla in 1, and pancreatic neuroendocrine tumor in 1). Operative procedures included pancreatectomies in 50 patients (PD in 42, distal pancreatectomy in 6, and total pancreatectomy in 2), hepatectomies in 13 (major hepatectomy in 6, anatomical sectionectomy in 4, anatomical subsectionectomy in 3), and 3 other procedures (transhepatic hilar bile duct resection in 1, common bile duct resection for congenital biliary disease in 1, and living donor liver transplant in 1).

**Table 1:** Microbiological data from gastric juice after general anesthesia induction

Positive culture in gastric juice	Group NGC *n* = 45	Group GC *n* = 21	*p* value
Fungus species	0	21 (100%)	
* Candida albicans*	0	15 (71.4%)	< 0.001
* Candida glabrata*	0	8 (38.1%)	< 0.001
* Candida tropicalis*	0	1 (4.8%)	0.318
Bacterial species	26 (57.8%)	13 (61.9%)	0.751
Gram-positive species			
* Streptococcus* species	19 (42.2%)	2 (9.5%)	0.008
* Enterococcus* species	4 (8.9%)	6 (28.6%)	0.047
* Staphylococcus* species	8 (17.8%)	2 (9.5%)	0.318
* Corynebacterium* species	6 (13.3%)	3 (14.3%)	0.596
* Bacillus* species	2 (4.4%)	0	0.462
* Lactobacillus* species	1 (2.2%)	0	0.682
Gram-negative species			
* Neisseria* species	7 (15.6%)	1 (4.8%)	0.203
* Escherichia coli*	0	2 (9.5%)	0.098
* Enterobacter* species	0	4 (19.0%)	0.008
* Klebsiella* species	0	1 (4.8%)	0.318
* Pseudomonas aeruginosa*	0	1 (4.8%)	0.318
* Morganella morganii*	0	1 (4.8%)	0.318
* Raoultella planticola*	1 (2.2%)	0	0.682

*NGC* no gastric Candida colonization, *GC* gastric Candida colonization

Positive rate: %

Fungal and bacterial species are overlapped

### Microbiological characteristics

**Fig. 1:**
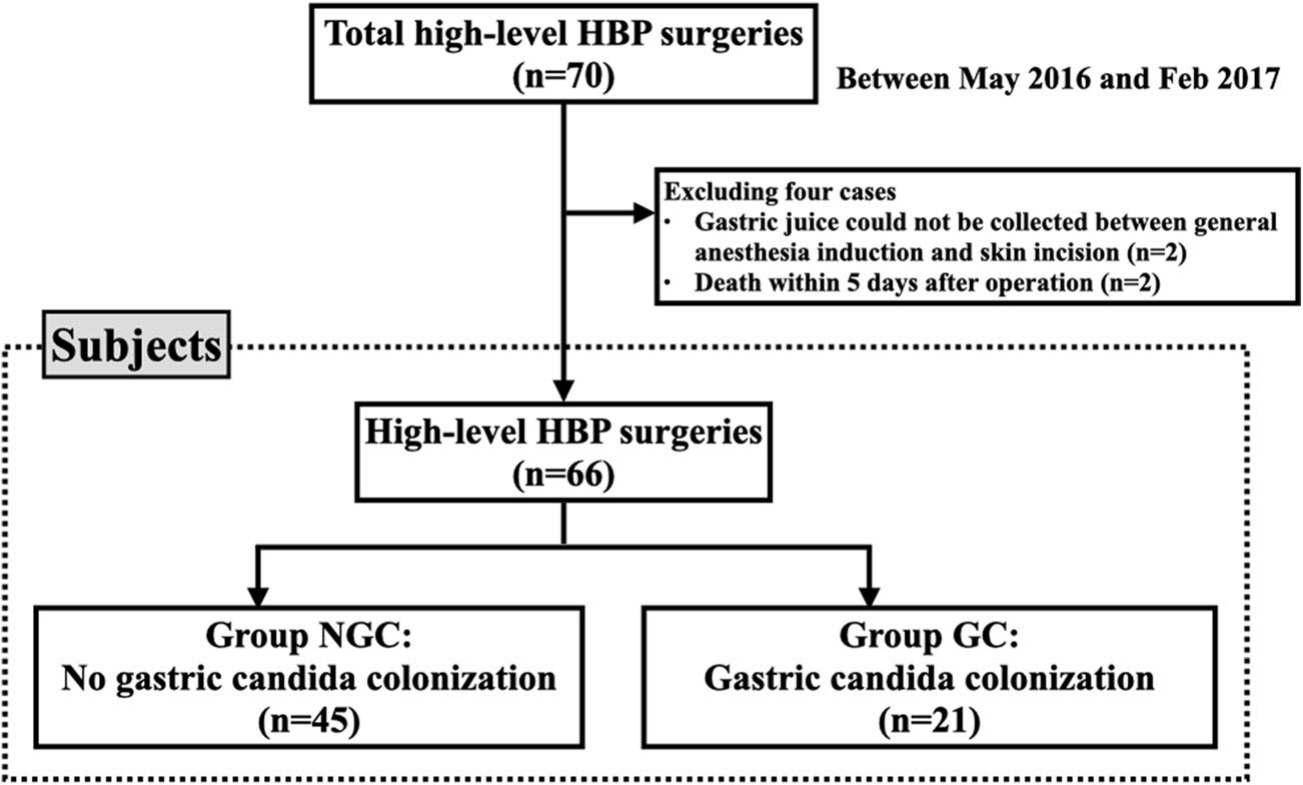
Patient distribution for the present study. Between May 2016 and February 2017, 70 patients were registered for this prospective study. After excluding four patients, the 66 patients were divided into two groups according to the presence of gastric Candida colonization. High-level hepatobiliary pancreatic (HBP) surgeries: pancreaticoduodenectomy, distal pancreatectomy for pancreatic ductal adenocarcinoma, total pancreatectomy, major hepatectomy of 3 segments or more, anatomical sectionectomy and subsectionectomy, common bile duct resection for congenital biliary disease, and liver transplantation according to the definition of the Japanese Society of Hepato-Biliary-Pancreatic Surgery

The 66 patients were classified into groups NGC (45 patients) and GC (21 patients) (Fig. 1). In the 45 patients of group NGC, no growth of *Candida* species was identified in 26 patients and the less than one-quadrant growth was identified in 19 patients.

Microbiological data of fungal and bacterial species in gastric juice is shown in Table 1. In group GC, *Candida albicans* was the most frequently identified *Candida* species: 71.4% (15/21), followed by *Candida glabrata* in 38.1% (8/21). Bacterial species were identified in 13 patients (61.9%). There was no significant difference in the detection rates of bacterial species from gastric juice between groups NGC and GC (57.8% vs. 61.9%, *p* = 0.751).

### Patient characteristics according to the presence of gastric Candida colonization

Preoperative variables were compared between groups NGC and GC (Table 2). There were no significant differences between the two groups in variables including inflammatory and nutritional markers such as NLR and prognostic nutritional index (PNI), lifestyle diseases like diabetes mellitus, alcohol consumption, smoking, biliary drainage, PS, cachexia, administration of PPI or H2 blocker (Supplemental Table 1a), the therapeutic use of a glucocorticoid, chemoradiotherapy, history of broad-spectrum antibiotics, types of primary disease, and previous malignant disease. Furthermore, multivariate analysis using preoperative variables which showed a *p* value of less than 0.25 (gender, cachexia, BI > 400, administration of PPI or H2 receptor blocker, biliary drainage, history of broad-spectrum antibiotics, chemoradiotherapy, briary drainage, malignancy/benign, primary disease, and PNI) in univariate analysis revealed no preoperative risk factors for gastric Candida colonization.

**Table 2:** Patient characteristics according to the presence of gastric Candida colonization

Variables	Group NGC *n* = 45	Group GC *n* = 21	*p* value
Preoperative variables			
Age, years	69 [23–91]	69 [49–82]	0.526
Gender, male/female	31/14 (68.9%)	10/11 (47.6%)	0.097
BMI (kg/m^2^)	20.8 [15.2–31.4]	21.5 [16.3–27.2]	0.980
Performance status: 0/1	39/6	17/4	0.396
Cachexia (yes/no)	9/36 (20.0%)	7/14 (33.3%)	0.239
Diabetes mellitus (yes/no)	12/33 (26.7%)	5/16 (23.8%)	0.805
Hypertension (yes/no)	20/25 (44.4%)	9/12 (42.9%)	0.904
Dyslipidemia (yes/no)	14/31 (31.1%)	6/15 (28.6%)	0.834
Smoking: Brinkman index > 400 (yes/no)	20/25 (44.4%)	6/15 (28.6%)	0.219
Alcohol consumption > 60 g/day (yes/no)	8/37 (17.8%)	2/19 (9.5%)	0.318
History of other malignant disease (yes/no)	9/36 (20.0%)	6/15 (28.6%)	0.318
Administration of PPI or H2 receptor blocker (yes/no)	23/22 (51.1%)	15/6 (71.4%)	0.120
Therapeutic use of glucocorticoid (yes/no)	2/43 (4.4%)	0/21 (0.0%)	0.462
Total parenteral nutrition (yes/no)	1/44 (2.2%)	0/21 (0.0%)	0.682
Biliary drainage (yes/no)	15/30 (33.3%)	12/9 (57.1%)	0.067
History of broad-spectrum antibiotics (yes/no)	8/37 (17.8%)	8/13 (38.1%)	0.073
Chemotherapy (yes/no)	10/35 (22.2%)	6/15 (28.6%)	0.575
Chemoradiotherapy (yes/no)	11/34 (24.4%)	10/11 (47.6%)	0.060
Primary disease (malignancy/benign)	34/11 (75.6%)	19/2 (90.5%)	0.137
Diagnosis			
PDAC/IPMN/BDC/HCC/others	16/7/7/6/9	14/1/3/0/3	0.112
Albumin (g/dl)	3.7 [3.0–4.6]	3.7 [2.9–4.9]	0.326
White blood cell counts (/mm^2^)	5000 [2580–13,290]	4880 [2480–7690]	0.259
Hemoglobin (g/dl)	12.3 [8.4–17.5]	11.7 [8.7–15.8]	0.237
Platelet counts (× 10^3^ ml)	197 [110–737]	200 [68–340]	0.736
C-reactive protein (mg/l)	0.12 [0.01–4.06]	0.12 [0.03–3.78]	0.929
NLR	2.52 [0.89–13.5]	3.00 [0.90–5.04]	0.397
PNI	45.4 [33.4–56.0]	41.5 [36.4–55.9]	0.100
Intraoperative variables			
Operation procedure Pancreaticoduodenectomy/other pancreatectomy/hepatectomy/others	26/5/11/3	16/3/2/0	0.272
Operative duration (min)	446 [198–972]	541 [348–880]	0.012
Blood loss (g)	566 [10–6017]	716 [151–4386]	0.256
Blood transfusion (yes/no)	18/27 (40.0%)	10/11 (47.6%)	0.560

Positive rate: % [range]

*BMI* body mass index, *PPI* proton pump inhibitor, *H2* histamine-2, *PDAC* pancreatic ductal adenocarcinoma, *IPMN* intraductal papillary mutinous neoplasm, *BDC* bile duct cancer, *HCC* hepatocellular carcinoma, *NLR* neutrophil/lymphocyte ratio, *PNI* prognostic nutritional index

### Comparisons of postoperative infectious complications between groups NGC and GC

Table 3 shows the comparison of surgical outcomes between NGC and GC. The incidence of SSIs was significantly higher in group GC than in group NGC: 57.1% (12/21) vs. 17.8% (8/45), *p* = 0.001. The incidence of organ or space SSIs was significantly higher in group GC than in group NGC: 52.4% (11/21) vs. 17.8% (8/45), *p* = 0.004. C-D grade of IIIa or more occurred significantly higher in group GC than in group NGC: 52.4% (11/21) vs. 22.2% (10/45), *p* = 0.014. Their main causes were pancreatic fistula, intra-abdominal abscess, and massive ascites, which were treated by drain management or percutaneous drainage. The duration of hospital stay after the operation was significantly higher in group GC than in group NGC: 32 days (18–62) vs. 24 days (10–74), *p* = 0.024. Readmission rates were also higher in group GC than in group NGC: 54.2% vs. 15.6%, *p* = 0.002. The main causes of readmission were cholangitis, liver abscess, and malnutrition.

**Table 3:** Postoperative complications according the presence of gastric Candida colonization

Postoperative complications		Group NGC *n* = 45	Group GC *n* = 21	*p* value
SSIs (yes/no)		8/37 (17.8%)	12/9 (57.1%)	0.001
Incisional SSIs (yes/no)		0/45 (0%)	1/20 (4.8%)	0.318
Organ or space SSIs (yes/no)		8/37 (17.8%)	11/10 (52.4%)	0.004
Postoperative complications of C-D ≥ IIIa (yes/no)		10/35 (22.2%)	11/10 (52.4%)	0.014
IIIa	Pancreatic fistula	3	2	0.513
	Unknown cause abscess	3	4	0.138
	Biliary fistula	1	1	0.538
	Massive ascites	0	1	0.318
	Hepaticojejunostomy stricture	1	0	0.682
IIIb	Anastomotic stenosis of portal vein^†^	1	1	0.538
IVa	Respiratory dysfunction	0	1	0.318
IVb	Respiratory and renal dysfunction	1	1	0.538
Duration of ICU stay, days		1 [1–20]	1 [1–6]	0.753
Duration of hospital stay after operation, days		24 [10–74]	32 [18–68]	0.024
Readmission, yes/no (%)		7/38 (15.6%)	11/10 (52.4%)	0.002
Cholangitis		3	4	0.138
Liver abscess		0	2	0.098
Malnutrition		0	2	0.098
Others		4	3	0.393

*NGC* no gastric Candida colonization, *GC* gastric Candida colonization, *SSIs* surgical site infections, *C-D* Clavien–Dindo classification

^†^Portal vein stenosis associated with periportal vein inflammation (positive rate: %) [range]

To investigate whether the influence of gastric Candida colonization differed or not, according to the surgical procedures, subgroup analysis was conducted (Supplemental Table 2). In 50 patients with enterotomy (same patient with hepaticojejunostomy), the incidents of SSIs were significantly higher in group GC than in group NGC: 50.0% (9/18) vs. 12.5% (4/32), *p* = 0.006. In the other 16 patients without enterotomy, the incidents of SSIs were higher in group GC than in group NGC: 100% (3/3) vs. 30.8% (4/13), *p* = 0.029. In 42 patients with PD and 24 patients with non-PD, the incidents of SSIs were significantly higher in group GC than in group NGC: 50.0% (8/16) vs. 11.5% (3/26), *p* = 0.009, and 26.3% (5/19) vs. 80.0% (4/5), *p* = 0.027, respectively.

There was no microbiological association between gastric juice and SSIs in the patients who developed SSIs (Table 4). In group NGC, microbiological culture of infected sites was carried out in seven patients of eight who developed SSIs. No bacterial species were identified in 42.9% (3/7) of subjects: pancreatic fistula after pancreatectomy (*n* = 2) and intra-abdominal abscess after hepatectomy (*n* = 1). The growth of *Candida* species was not identified in infected sites. In group GC, culture of infected sites was carried out in 11 patients among the 12 who developed SSIs. No bacterial species were identified in one subject: pancreatic fistula after pancreatectomy. Three patients preoperatively had *Candida glabrata* colonization in their gastric juice and postoperatively developed *Candia glabrata* infection in SSIs: biliary fistula (*n* = 1), massive ascites (*n* = 1), and intra-abdominal abscess (*n* = 1).

**Table 4:** The microbiological data of patients who developed SSIs

*N*	Op	Enterotomy	Gastric juice	SSIs		
				Infected sites	POD	Microbiological species
Group NGC						
1	Hep	No	*Streptococcus* spp., CNS	IAA	7	*Enterobacter asburiae*
2	Hep	Yes	Negative	BF	8	*Enterococcus faecalis*, *Enterococcus fecium*, CNS
3	DP	No	Negative	PF	10	MRSA, CNS
4	DP	No	a-, r-*Streptococcus*	PF	7	NA
5	SSPPD	Yes	*Bacillus* species	PF	11	Negative
6	SSPPD	Yes	Negative	IAA	7	*Enterococcus faecalis*
7	Hep	No	a-, r-*Streptococcus*, *Neisseria* species	IAA	14	Negative
8	SSPPD	Yes	Negative	PF	6	Negative
Group GC						
1	Hep	No	Ca	IAA	6	CNS
2	SSPPD	Yes	Ca	IAA	12	CNS
3	SSPPD	Yes	Ca, Cg	BF	3	Cg, *Enterobacter* spp., *Enterococcus faecalis*
4	SSPPD	Yes	Ca, Cg, *Escherichia coli*	Ascites	6	Cg, *Bacillus cereus*
5	SSPPD	Yes	Ca	Wound	7	*Enterococcus faecalis*, *Enterococcus avium*
6	SSPPD	Yes	Ca, *Pseudomonas aeruginosa*, *Enterobacter* spp., *Escherichia coli*	PF	6	NA
7	DP	No	Cg, *Klebsiella pneumoniae*	PF	11	MSSA, *Enterococcus faecalis*, coryneform bacteria
8	SSPPD	Yes	Ca, *Morganella morganii*	Ascites	6	*Enterococcus faecalis*, *Pseudomonas aeruginosa*
9	Hep	Yes	Cg	IAA	10	*Enterococcus faecalis*, *Enterobacter aerogenes*
10	SSPPD	Yes	Cg, coryneform bacteria	IAA	9	Cg, *Leuconostoc pseudomesenteroides*, *Citrobacter farmeri*, *Klebsiella oxytoca*, *Fusobacterium varium*
11	DP	No	Ca, *Streptococcus* spp., *Neisseria* spp.	PF	10	Negative
12	SSPPD	Yes	Ca, *Enterococcus faecalis*, coryneform bacteria	IAA	14	*Enterococcus faecalis*

*NGC* no gastric Candida colonization, *GC* gastric Candida colonization, *SSIs* surgical site infections, *Op* operation, *POD* postoperative day, *spp.* species, *SSPPD* subtotal stomach-preserving pancreaticoduodenectomy, *DP* distal pancreatectomy, *Hep* hepatectomy, *Ca Candida albicans*, *Cg Candida glabrata*, *CNS* coagulase-negative staphylococcus, *MRSA* methicillin-resistant *Staphylococcus aureus*, *MSSA* methicillin-susceptible *Staphylococcus aureus*, *PF* pancreatic fistula, *BF* biliary fistula, *IAA* intra-abdominal abscess, *NA* not available for drainage of infected sites

### Uni- and multivariate analyses for identifying pre- and intraoperative variables associated with SSIs

Univariate analysis revealed gastric Candida colonization as a significant risk factor of SSIs (*p* = 0.001). Multivariate analysis also identified gastric Candida colonization as a significant independent risk factor of SSIs (Odds ratio 6.17, *p* = 0.002) (Table 5).

**Table 5:** Uni- and multivariate analyses for identifying the risk factors of SSIs

Variables	Univariate			Multivariate		
	SSIs no *n* = 46	SSIs yes *n* = 20	*p* value	Odds ratio	95% CI	*p* value
Preoperative variables						
Age (years)	69 [23–91]	69 [32–83]	0.328			
Gender (male/female)	30/16 (65.2%)	11/9 (55.0%)	0.432			
BMI (kg/m^2^)	20.7 [15.2–31.4]	22.0 [18.1–27.3]	0.276			
Performance status: 0/1	40/6	16/4	0.352			
Cachexia (yes/no)	10/36 (21.7%)	6/14 (30.0%)	0.336			
Diabetes mellitus (yes/no)	13/33 (28.3%)	4/16 (20.0%)	0.481			
Hypertension (yes/no)	20/26 (43.5%)	9/11 (45.0%)	0.909			
Dyslipidemia (yes/no)	15/31 (32.6%)	5/15 (25.0%)	0.536			
Smoking: Brinkman index > 400 (yes/no)	16/30 (34.8%)	10/10 (50.0%)	0.245			
Alcohol consumption > 60 g/day (yes/no)	8/38 (17.4%)	2/18 (10.0%)	0.359			
Past history of malignant disease (yes/no)	11/35 (23.9%)	4/16 (20.0%)	0.498			
Administration of PPI or H2 receptor blocker (yes/no)	27/19 (58.7%)	11/9 (55.0%)	0.780			
Therapeutic use of glucocorticoid (yes/no)	2/44 (4.3%)	0/20 (0%)	0.483			
Total parenteral nutrition (yes/no)	0/46 (0.0%)	1/19 (5.00%)	0.303			
Biliary drainage (yes/no)	19/27 (41.3%)	8/12 (40.0%)	0.921			
History of broad-spectrum antibiotics (yes/no)	10/36 (21.7%)	6/14 (30.0%)	0.336			
Chemotherapy (yes/no)	13/33 (28.3%)	3/17 (15.0%)	0.202			
Chemoradiotherapy (yes/no)	15/31 (32.6%)	6/14 (30.0%)	0.834			
Primary disease (malignancy/benign)	37/9 (80.4%)	16/4 (80.0%)	0.606			
Diagnosis PDAC/IPMN/BDC/HCC/others	21/6/6/5/8	9/2/4/1/4	0.889			
Gastric Candida colonization (yes/no)	9/37 (19.6%)	12/8 (60.0%)	0.001	6.17	1.95–19.5	0.002
Gastric bacterial colonization	27/19 (58.7%)	12/8 (60.0%)	0.921			
Albumin (g/dl)	3.7 [3.0–4.6]	3.9 [2.9–4.9]	0.713			
White blood cell counts (/mm2)	4955 [2480–13,290]	5070 [2670–8170]	0.873			
Hemoglobin (g/dl)	12.1 [8.4–17.5]	11.9 [8.8–15.8]	0.564			
Platelet counts (× 10^3^ ml)	197 [96–737]	212 [68–340]	0.596			
C-reactive protein (mg/l)	0.12 [0.01–4.06]	0.13 [0.01–3.78]	0.451			
NLR	2.83 [1.02–13.5]	2.04 [0.89–5.04]	0.084			
PNI	43.2 [36.3–56.0]	45.6 [33.4–56.0]	0.337			
Intraoperative variables						
Operation procedure Pancreaticoduodenectomy/other pancreatectomy Hepatectomy/others	31/4/8/3	11/4/5/0	0.318			
Operative duration (min)	459 [198–972]	494 [244–880]	0.831			
Blood loss (g)	589 [54–6017]	679 [10–4386]	0.967			
Blood transfusion (yes/no)	18/28 (39.1%)	10/10 (50.0%)	0.412			

Positive rate:% [range]

*PPI* proton pump inhibitor, *H2* histamine-2, *PDAC* pancreatic ductal adenocarcinoma, *IPMN* intraductal papillary mutinous neoplasm, *BDC* bile duct cancer, *HCC* hepatocellular carcinoma, *BMI* body mass index, *NLR* neutrophil/lymphocyte ratio, *PNI* prognostic nutritional index

## Discussion

In the present study, we newly elucidated the following insights: (1) Asymptomatic gastric Candida colonization is preoperatively found in approximately 30% of patients for whom high-level HBP surgery was proposed. (2) In such patients, the risk of SSIs and postoperative morbidity was notably higher after high-level HBP surgery. (3) These results were consistent regardless of the type of procedure (with or without enterotomy and PD or non-PD).

*Candida* species are commonly considered to colonize the human gut as a component of the resident microbiota. Their presence is usually regarded as benign. However, high-level Candida colonization is reported to be associated with gastrointestinal tract diseases including Crohn’s disease, ulcerative colitis, and gastric ulcer [25–27]. However, there have been no studies that prospectively assess the fungal and bacterial flora of the stomach, and this is the first study focusing on the resident microbiota in the stomach and the association between Candida colonization and SSIs after HBP surgeries.

The rate of Candida infection tends to increase with increasing age and is significantly high especially in those with age over 60 because their immune systems are weaker and their participation in outdoor activities is not low [28]. In critically ill patients recovering from abdominal surgery, 5–10% of them have a probability of developing invasive Candida infection of candidemia and Candida peritonitis by exposure to an increasing amount of *Candida* species in non-functioning bowel, prolonged antibiotic therapy, and organ support of central venous catheters, intubation tubes, Foley catheters, and nasogastric tubes [13, 14]. Therefore, we hypothesized that patients with compromising factors like malignant disease, diabetes mellitus, or poor nutritional status had asymptomatic fungal colonization of the digestive tract, and therefore highly developed postoperative infectious complications. In the present study, however, gastric Candida colonization was not related to patients’ compromised status such as age, PS, cachexia, primary disease, preoperative biliary drainage, and chemoradiotherapy, but was a unique significant risk factor of SSIs. According to previous reports, gastrointestinal Candida colonization is linked to poor oral hygiene and a western diet with increased consumption of purified wheat flour in healthy people [29–32]. *Candida* species colonized preoperatively in the gastrointestinal tract might increase after invasive procedures of high-level HBP surgeries and might cause infectious complications.

High-level HBP surgeries continue to be accompanied by postoperative complications despite the improvement in surgical techniques and perioperative care. The morbidity following PD ranges from 30 to 70%, and postoperative mortality rates remain approximately between 1 and 5% even in high-volume centers [3–5]. Major hepatectomy also has high morbidity rates of 24–70%, and perioperative mortality rates range from 1.8 to 8.4% [1, 6]. In such clinical courses, complications are highly associated with infection by microorganisms. Preoperative biliary drainage and instrumentations are associated with postoperative infectious complications of wound infection and intra-abdominal abscess, and microorganisms in the bile show a concordance of 100% and 69% of microorganisms in intra-abdominal abscess and wound infection [7, 8]. Therefore, perioperative use of specific antibiotics based on bile culture is recommended for preventing infectious complications [9], whereas few studies are focusing on SSI and fungal infection. Kato et al. [10, 11] reported a negative impact of biliary candidiasis on postoperative complications after PD. A total of 66.7% of patients with biliary candidiasis developed an intra-abdominal abscess, 14.3% sepsis, 14.3% PV thrombosis, and 9.5% abdominal hemorrhage. Moreover, the incidence of leakage in pancreatojejunostomy (grade B or C) was significantly higher in patients with biliary candidiasis compared with those without it (38.1% vs. 5.7%, *p* = 0.019). In the present study, a highly significant number of the patients with gastric Candida colonization experienced postoperative complications of organ or space SSIs, C-D grade of IIIa or higher, prolonged hospital stays, and readmission, compared with those without gastric Candida colonization in high-level HBP surgeries. Candida colonization is a significant issue to be addressed for decreasing morbidity and mortality rates in HBP surgery fields, although it remains underestimated as compared with other infectious diseases [33, 34].

The underlining mechanism that gastric Candida colonization increases SSIs regardless of the operative procedures has yet to be addressed. *Candida* species in gastric juice might develop contamination of the peritoneum by opening bowel flora, invasion into tissues from mucosal defects, and translocation into pancreatic juice or bile juice, causing the serious complications of postoperative massive ascites, pancreatic fistula, biliary fistula, and cholangitis. According to previous studies, fungal–bacterial coinfection might be associated with SSIs. *Candida albicans* initially adheres to and invades an epithelial cell, causing fungal recognition and the formation of hyphae. Hypha formation causes epithelial cell damage and activates the immune system through the secretion of a cytolytic peptide toxin called Candidalysin. Activated epithelial cells lead to the production of proinflammatory cytokines, chemokines, and antimicrobial peptides to recruit neutrophil and macrophage [35, 36]. The organ wall damage caused by *Candida* species allows bacteria to penetrate more easily in mouse models [37, 38]. Furthermore, *Candida albicans* produces a biofilm of a heterogeneous mixture of other microbial species and directly stimulates the growth of the species in vitro, causing resistance to antibiotic therapy [37]. In the current study, the same *Candida* species were identified in 27.3% of specimens from infected sites in group GC, whereas *Candida* species were not identified from infected sites in group NGC. Bacteria detection rates from infected sites tended to be higher in group GC than in group NGC although there was no significant difference. The results of the subgroup analysis show that the incidence of SSIs was significantly higher not only in the GC group receiving surgery accompanied by enterotomy but also in those without enterotomy, suggesting that *Candida* species in gastric juice not only cause direct damage to the organs but also accelerate other bacterial infections.

In the present study, the concordance rate of microorganisms between gastric juice and infected sites was low. *Candida* species can proliferate in the stomach by adjusting the thickness of the outer cell wall layer [39]. However, the majority of bacterial species are influenced by low pH in the stomach [40]. Major SSIs in high-level HBP surgeries were pancreatic fistula, biliary fistula, and intra-abdominal abscess. In such situations, bacterial species in the intestinal tract are different from those in gastric juice and might play a significant role in the development of SSIs, although *Candida* species can survive in the gastrointestinal tract. Preoperative biliary drainage, which is widely accepted as a risk factor of postoperative complications, had no association with SSIs. In cases with preoperative biliary drainage, perioperative antibiotics were selected based on the culture of the bile obtained by the drainage. We consider that such perioperative management contributed to decreasing the influence of preoperative biliary drainage on SSIs.

The potential limitation of this study is the small case number, one institutional and observational study. Therefore, the results might be confounded by institution-specific factors such as surgical technique and postoperative management. Moreover, we did not perform and validate *Candida*’s real-time polymerase chain reaction tests to evaluate *Candida* colonization in gastric juice and SSIs. To confirm the legitimacy of our results, multi-institutional prospective surveillance would be required. Currently, we propose a randomized control study (preoperative Candida eradication vs. no treatment) to apply our results to the clinical setting.

In conclusion, gastric Candida colonization is highly associated with SSIs after high-level HBP surgeries. Therefore, preoperative gastric Candida detection could be useful for predicting the development of SSIs after high-level HBP surgery, allowing us to pay maximum attention to SSIs and other complications.

## Electronic supplementary material

ESM 1 (PDF 57 kb)

ESM 2 (PDF 62 kb)
