# Supplementary material for: Association between gastric Candida colonization and surgical site infections after high-level hepatobiliary pancreatic surgeries: the results of prospective observational study
Source: Langenbecks Arch Surg. 2020 Oct 15;406(1):109–19. doi: 10.1007/s00423-020-02006-7 (PMC7870610; doi:10.1007/s00423-020-02006-7)
Supplement: Supplementary file 1 — (PDF 57 kb) [file 423_2020_2006_MOESM1_ESM.pdf]

Supplemental Table 1a. The influence of dose of proton pump inhibitor and H2 blocker on gastric candida colonization

PPI	Group NGC with PPI (n=21)	Group GC with PPI (n=13)	p-value
High dosage/standard dosage	12/9	6/7	0.533

H2 receptor blocker	Group NGC with PPI (n=2)	Group GC with PPI (n=2)	p-value
High dosage/standard dosage	2/0	2/0	-

NGC: no gastric candida colonization, GC: gastric candida colonization, PPI: proton pump inhibitor, H2: histamine-2

Supplemental Table 1b. The influence of chemotherapy regimen on gastric candida colonization

Chemotherapy	Group NGC with chemotherapy (n=10)	Group GC with chemotherapy (n=6)	p-value
GS/GC/Others	6/2/2	5/0/1	0.468

NGC: no gastric candida colonization, GC: gastric candida colonization, G: gemcitabine, GS: gemcitabine and S-1 combination therapy, GC: gemcitabine and cysplatin therapy

Association between gastric candida colonization and surgical site infections after high-level hepatobiliary pancreatic surgeries: the results of prospective observational study.

“Langenbeck's Archives of Surgery”

Kazuyuki Gyoten, Hiroyuki Kato, Aoi Hayasaki, Takehiro Fujii, Yusuke Iizawa, Yasuhiro Murata, Akihiro Tanemura, Naohisa Kuriyama, Masashi Kishiwada, Shugo Mizuno, Masanobu Usui, Hiroyuki Sakurai, Shuji Isaji.

Department of Hepatobiliary Pancreatic and Transplant Surgery, Mie University School of Medicine, Tsu, Mie, Japan.

Corresponding author: Hiroyuki Kato, E-mail: katohiroyuki510719@gmail.com
