# Supplementary material for: Association between gastric Candida colonization and surgical site infections after high-level hepatobiliary pancreatic surgeries: the results of prospective observational study
Source: Langenbecks Arch Surg. 2020 Oct 15;406(1):109–19. doi: 10.1007/s00423-020-02006-7 (PMC7870610; doi:10.1007/s00423-020-02006-7)
Supplement: Supplementary file 2 — (PDF 62 kb) [file 423_2020_2006_MOESM2_ESM.pdf]

Supplemental Table 2. The influence of gastric candida colonization on SSIs according to operative procedures

High-level HBP surgeries with enterotomy (n=50)	Group NGC (n=32)	Group GC (n=18)	p-value
SSIs (yes/no)	4/28 (12.5 %)	9/9 (50.0 %)	**0.006**

High-level HBP surgeries without enterotomy (n=16)	Group NGC (n=13)	Group GC (n=3)	p-value
SSIs (yes/no)	**4/9 (30.8 %)**	**3/0 (100 %)**	**0.029**

Pancreaticoduodenectomy (n=42)	Group NGC (n=26)	Group GC (n=16)	p-value
SSIs (yes/no)	3/23 (11.5 %)	8/8 (50.0 %)	**0.009**

Non-pancreaticoduodenectomy (n=24)	Group NGC (n=19)	Group GC (n=5)	p-value
SSIs (yes/no)	5/14 (26.3 %)	4/1 (80.0 %)	**0.027**

NGC: no gastric candida colonization, GC: gastric candida colonization, HBP: hepatobiliary pancreatic, SSIs: surgical site infections, PPI: proton pump inhibitor, H2: histamine-2

Association between gastric candida colonization and surgical site infections after high-level hepatobiliary pancreatic surgeries: the results of prospective observational study.

“Langenbeck's Archives of Surgery”

Kazuyuki Gyoten, Hiroyuki Kato, Aoi Hayasaki, Takehiro Fujii, Yusuke Iizawa, Yasuhiro Murata, Akihiro Tanemura, Naohisa Kuriyama, Masashi Kishiwada, Shugo Mizuno, Masanobu Usui, Hiroyuki Sakurai, Shuji Isaji.

Department of Hepatobiliary Pancreatic and Transplant Surgery, Mie University School of Medicine, Tsu, Mie, Japan.

Corresponding author: Hiroyuki Kato, E-mail: katohiroyuki510719@gmail.com
